# Effects of internet addiction and academic satisfaction on mental health among college students after the lifting of COVID-19 restrictions in China

**DOI:** 10.3389/fpsyt.2023.1243619

**Published:** 2023-10-26

**Authors:** Ai-Ping Deng, Cong Wang, Jia Cai, Zhong-Yue Deng, Yun-Fei Mu, Hong-Jun Song, Ya-Jing Meng, Xian-Dong Meng, Xue-Hua Huang, Lan Zhang, Yi Huang, Wei Zhang, Jin Chen, Mao-Sheng Ran

**Affiliations:** ^1^Mental Health Center, West China Hospital, Sichuan University, Chengdu, Sichuan, China; ^2^West China School of Nursing, Sichuan University, Chengdu, Sichuan, China; ^3^Psychiatric Laboratory, West China Hospital, Sichuan University, Chengdu, Sichuan, China; ^4^Department of Clinical Epidemiology and Evidence-Based Medicine, West China Hospital, Sichuan University, Chengdu, Sichuan, China

**Keywords:** academic satisfactory, China, internet addiction, mental health, students

## Abstract

**Background:**

Internet addiction (IA) among students, worsened by Coronavirus disease 2019 (COVID-19) pandemic, has become a social problem with the digitalization of school learning and many aspects of daily life. However, few studies on IA have been conducted among students after the lifting of COVID-19 restrictions in China.

**Method:**

This large-sample, cross-sectional, online survey was conducted to explore the characteristics of IA and the association among IA, academic satisfaction, and mental health problems from December 14, 2022 to February 28, 2023 in Sichuan, China. All participants (*N* = 22,605) were students in colleges and universities, recruited via their teachers and professors.

**Results:**

Of all the participants, 14,921 (66.0%) participants had IA. Participants with IA were more likely to have depression symptom, anxiety symptom, insomnia, and lifetime suicidal ideation. In addition, participants with severe IA had significantly higher rates of mental health problems (e.g., depression, anxiety, insomnia, and suicidal ideation) than those with mild IA. A significant IA-by-academic satisfactory-interaction on mental health was identified: participants with higher level of IA showed particularly severe symptom of depression, anxiety and insomnia when affected by low satisfactory of academy (*p* < 0.001).

**Conclusion:**

This study reveals that IA has a significantly negative impact on mental health among college students after the lifting of COVID-19 restrictions in China. IA and academic satisfaction have interactive impacts on mental health problems among students. Further educational and health policies and psychosocial interventions should be developed to reduce IA and enhance academic satisfaction for improving students’ mental health.

## 1. Introduction

Internet use has snowballed with its gravity (essentiality and availability) in the lives of modern students. While older adults are also utilizing the internet more frequently, the majority of internet users are adolescents and young people, particularly among the digital generation (1–4). Research indicates that an imbalance between internet use and sleep will directly impact academic performance and long-term physical and mental health (5, 6).

The prevalence of internet addiction (IA) varies among different populations. Previous studies have reported the prevalence of IA ranging from 0.5 to 84% (7–10). In China, the prevalence of IA was found to be between 0.9 and 37.9% among adolescents and young adults (7, 9, 11, 12). Medical students exhibited a relatively higher rate of IA, reaching 41.9% (10). During the Coronavirus Disease 2019 (COVID-19) pandemic, evidence showed that 32.4% of Chinese students displayed a tendency towards IA (13). However, the prevalence of IA and its influencing factors among college students after the lifting of COVID-19 restrictions in China remains unknown.

Previous studies have showed the associations among IA, sleep problems, and mental health problems (14–17). A meta-analysis conducted in 2019, comprising 23 observational studies with 35,684 participants, revealed that individuals with IA had a pooled odds ratio of 2.2 for experiencing sleep problems and a 24% reduction in sleep duration compared to normal internet users (6). Furthermore, IA has been highly correlated with depression and anxiety (18–21), as well as poor academic performance (22, 23). However, further research is needed to explore the relationship between IA, academic performance, and mental health problems, particularly among college students after the lifting of COVID-19 restrictions in China.

Therefore, this study aimed to (1) investigate the prevalence of IA and (2) examine the association between IA, academic satisfaction, and mental health problems among college students in Sichuan, China, after the lifting of COVID-19 restrictions.

## 2. Methods

### 2.1. Study design and participants

This cross-sectional online study was conducted among college students from December 14, 2022, to February 28, 2023, in the period immediately after the lifting of COVID-9 related restrictions in Sichuan, China. The study was approved by the Biomedical Research Ethics Committee of West China Hospital, Sichuan University (No: 2022-1790). Written informed consent was obtained online before the survey.

A self-designed online survey was released via a platform of Wenjuanxing. In order to ensure the quality of the study, the survey information was firstly sent to the teachers and professors in colleges and universities in Sichuan province, China. Then, it was disseminated from teachers and professors directly to their students. The confidentiality of all data was ensured. The inclusion criteria of participants: (1) Students studying at a university or college in Sichuan Province and (2) Voluntarily agree to participate in this survey. The exclusion criteria: (1) Students in other Province other than Sichuan and (2) Do not agree to participate in this survey. A total of 22,605 university and college students provided informed consents and completed the questionnaires.

### 2.2. Measurements

The questionnaire had 4 parts, taking approximately 20 min to complete. The first part included demographic and general information of the participants, such as sex, ethnicity, monthly family income, history of psychotic disorders, etc. The second part collected the information related to the COVID-19 pandemic, including the COVID-19 infection, quarantine experience, and psychological stress level. The third part inquired the participants’ daily habits and behaviors. The fourth part investigated current mental health problems, including symptoms of depression, anxiety, insomnia, internet addiction and social support.

The Patient Health Questionnaire (PHQ-9) was used to assess the level of depressive symptoms in the past 2 weeks (24). The total score of greater than 5 indicating depression symptoms (25). The good reliability and validity have been established for the Chinese versions of PHQ-9, which has been commonly used for Chinese general population (25, 26).

The Generalized Anxiety Disorder-7 (GAD-7) was used to assess symptoms of anxiety (27), with a total score of greater than 5 indicating symptoms of anxiety (27). The Chinese versions of GAD-7 has good reliability and validity among Chinese people (28).

The Insomnia Severity Index (ISI) was used to assess insomnia (29), with a total score of greater than 8 indicating insomnia. The Chinese versions of ISI has good reliability and validity among Chinese people (30).

The Internet Addiction Test (IAT) was used to assess internet addiction (31), which comprises 20 items and each item is scored on a five-point scale. An IA level was classified as mild, moderate, or severe according to the total score of IAT. The cut off score of 40 was used to categorize as average usage group (<40), and problematic usage group (≥40). The good reliability and validity have been established for the Chinese version of IAT among Chinese adolescents (32).

The Social Support Rating Scale (SSRS) was adopted in the current study to measure social support (33), which includes 10 items with three dimensions: objective support, subjective support, and support utilization. A higher total score indicates a higher level of social support for the participants. The Chinese version of SSRS has good reliability and validity among Chinese people (33).

### 2.3. Statistical analysis

Chi-square test and two-sample *t*-test were used to compare demographic variables and scales for categorical and continuous variables as appropriate between the 2 groups. Two sample *t*-test and Chi-square test were performed to compare the score of PHQ-9, GAD-7, ISI, and SSRS, and the percentage of lifetime suicidal ideation between the 2 groups. Linear regression models with IA as a dependent variable and academic satisfactory as an independent variable was performed to examine the main effects of IA and academic satisfactory as well as their interaction on mental health problems (depression, anxiety and insomnia). All analyses controlled for demographic (i.e., age, sex, nationality, household registered residence, single child, family income, educational level of father/mother, smoking, drinking, family history of psychosis, history of psychotic disorder, marital status of parents, and parenting style) in order to adjust for potential variation in study variables attributed to these characteristics. SPSS (version 22.0) was used for data analysis. The threshold for statistical significance was set at 2-sided, *p* < 0.05.

## 3. Result

### 3.1. Demographic information

There were 22,605 college students finished the survey in total, which includes 7,684 participants (34%, age = 19.14 ± 1.224, Female = 64.9%) in the without IA group, and 11,969 participants (66%, age = 19.09 ± 1.198, Female = 64.9%) in the with IA group (Table 1). Compared with participants in the without IA group, participants in the with IA group had significantly higher percentage of Han nationality (87.8% vs. 89.3%), smoking (6.8% vs. 7.6%), drinking (15.4% vs. 20.1%), family history of psychosis (0.6% vs. 1.1%), history of psychotic disorder (4.1% vs. 6.9%), infection of COVID-19 (33.1% vs. 38.2%), and quarantine experience (34.8% vs. 38.7%) (*p* < 0.001). Participants in the with IA group had significantly lower percentage of progress (22% *VS* 15.7%) and higher retrogression (33.1% *VS* 50.8%) in academic performance and high percentage of academy unsatisfaction than those in the without IA group (7.1% *VS* 15.6%) (*p* < 0.001).

**Table 1:** Demographic characteristics of the participants.

	Total (*N* = 22,605)	Without internet addiction (*N* = 7,684)	With internet addiction (*N* = 14,921)	*p*
**Age**	19.1 ± 1.207	19.14 ± 1.224	19.09 ± 1.198	<0.05
**Sex**				>0.05
Male (%)	7,927 (35.1)	2,688 (35.0)	5,239 (35.1)	
Female (%)	14,678 (64.9)	4,996 (65.0)	9,682 (64.9)	
**Grade**				>0.05
Grade 1 (%)	13,958 (61.7)	4,710 (61.3)	9,248 (62.0)	
Grade 2 (%)	6,493 (28.7)	2,245 (29.2)	4,248 (28.5)	
Grade 3 (%)	1,845 (8.2)	631 (8.2)	1,214 (8.1)	
Grade 4 and 5 (%)	309 (1.4)	98 (1.3)	211 (1.4)	
**Nationality**				<0.001
Han (%)	20,078 (88.8)	6,750 (87.8)	13,328 (89.3)	
Other (%)	2,527 (11.2)	934 (12.2)	1,593 (10.7)	
**Registered residence**				>0.05
Rural (%)	17,921 (79.3)	6,108 (79.5)	11,813 (79.2)	
Urban (%)	4,684 (20.7)	1,576 (20.5)	3,108 (20.8)	
**Single-child**				>0.05
Yes (%)	5,455 (24.1)	1,885 (24.5)	3,570 (23.9)	
No (%)	17,150 (75.9)	5,799 (75.5)	11,351 (76.1)	
**Romantic relationship status**				>0.05
Single (%)	16,535 (73.1)	5,615 (73.1)	10,920 (73.2)	
Have a lover (%)	6,070 (26.9)	2,069 (26.9)	4,001 (26.8)	
**Family income (monthly)**				>0.05
<2040 RMB (%)	5,271 (23.3)	1,824 (23.7)	3,447 (23.1)	
2040–4,999 RMB (%)	8,931 (39.5)	3,096 (40.3)	5,835 (39.1)	
5,000–9,999 RMB (%)	5,572 (24.6)	1,843 (24.0)	3,729 (25.0)	
>10,000 RMB (%)	2,831 (12.5)	921 (12.0)	1,910 (12.8)	
**Education level of Father**				<0.05
Primary school and below (%)	7,729 (34.2)	2,687 (35.0)	5,042 (33.8)	
Middle school (%)	9,313 (41.2)	3,124 (40.7)	6,189 (41.5)	
High school (%)	3,638 (16.1)	1,262 (16.3)	2,376 (15.9)	
College and above (%)	1,925 (8.5)	611 (8.0)	1,314 (8.8)	
**Education level of Mother**				>0.05
Primary school and below (%)	10,110 (44.7)	3,465 (45.1)	6,645 (44.5)	
Middle school (%)	8,228 (36.4)	2,768 (36.0)	5,460 (36.6)	
High school (%)	2,943 (13.0)	1,002 (13.0)	1,941 (13.0)	
College and above (%)	1,324 (5.9)	449 (5.8)	875 (5.9)	
**Smoking**				<0.001
Yes (%)	1,654 (7.3)	523 (6.8)	1,131 (7.6)	
No (%)	20,951 (92.7)	7,161 (93.2)	13,790 (92.4)	
**Drinking**				<0.001
Yes (%)	4,177 (18.5)	1,180 (15.4)	2,997 (20.1)	
No (%)	18,428 (81.5)	6,504 (84.6)	11,924 (79.9)	
**Family history of psychosis**				<0.001
Positive (%)	217 (1.0)	46 (0.6)	171 (1.1)	
Negative (%)	22,388 (99.0)	7,638 (99.4)	14,750 (98.9)	
**History of psychotic disorder**				<0.001
Positive (%)	1,352 (6.0)	317 (4.1)	1,035 (6.9)	
Negative (%)	21,253 (94.0)	7,367 (95.9)	13,886 (93.1)	
**Marital status of parents**				>0.05
Unmarried (%)	381 (1.7)	113 (1.5)	268 (1.8)	
Married (%)	18,544 (82.0)	6,333 (82.4)	12,211 (81.8)	
Divorced (%)	1,929 (8.5)	649 (8.4)	1,280 (8.6)	
Remarried (%)	1,313 (5.8)	444 (5.8)	869 (5.8)	
Other (%)	438 (1.9)	145 (1.9)	293 (2.0)	
**Parenting style**				<0.001
Authoritative (%)	10,913 (48.3)	4,296 (55.9)	6,617 (44.3)	
Autocratic (%)	4,921 (21.8)	1,454 (18.9)	3,467 (23.2)	
Ignorant (%)	2,534 (11.2)	650 (8.5)	1,884 (12.6)	
Submissive (%)	4,237 (18.7)	1,284 (16.7)	2,953 (19.8)	
**Infection of COVID-19**				<0.001
Yes (%)	8,241 (36.5)	2,542 (33.1)	5,699 (38.2)	
No (%)	14,364 (63.5)	5,142 (66.9)	9,222 (61.8)	
**Quarantine experience**				<0.001
Yes (%)	8,446 (37.4)	2,676 (34.8)	5,770 (38.7)	
No (%)	14,159 (62.6)	5,008 (65.2)	9,151 (61.3)	
**Academic achievement**				<0.001
Progress	4,032 (17.8)	1,687 (22.0)	2,345 (15.7)	
Unaffected	8,456 (37.4)	3,456 (45.0)	5,000 (33.5)	
Retrogression	10,117 (44.8)	2,541 (33.1)	7,576 (50.8)	
**Academic satisfaction**				<0.001
Exceptional	1,561 (6.9)	744 (9.7)	817 (5.5)	
Good	4,310 (19.1)	2,044 (26.6)	2,266 (15.2)	
Satisfactory	13,282 (58.8)	4,267 (55.5)	9,015 (60.4)	
Unsatisfactory	2,871 (12.7)	542 (7.1)	2,329 (15.6)	
Very unsatisfactory	581 (2.6)	87 (1.1)	494 (3.3)	

### 3.2. Comparison of mental health problems between the two groups

**Figure 1:**
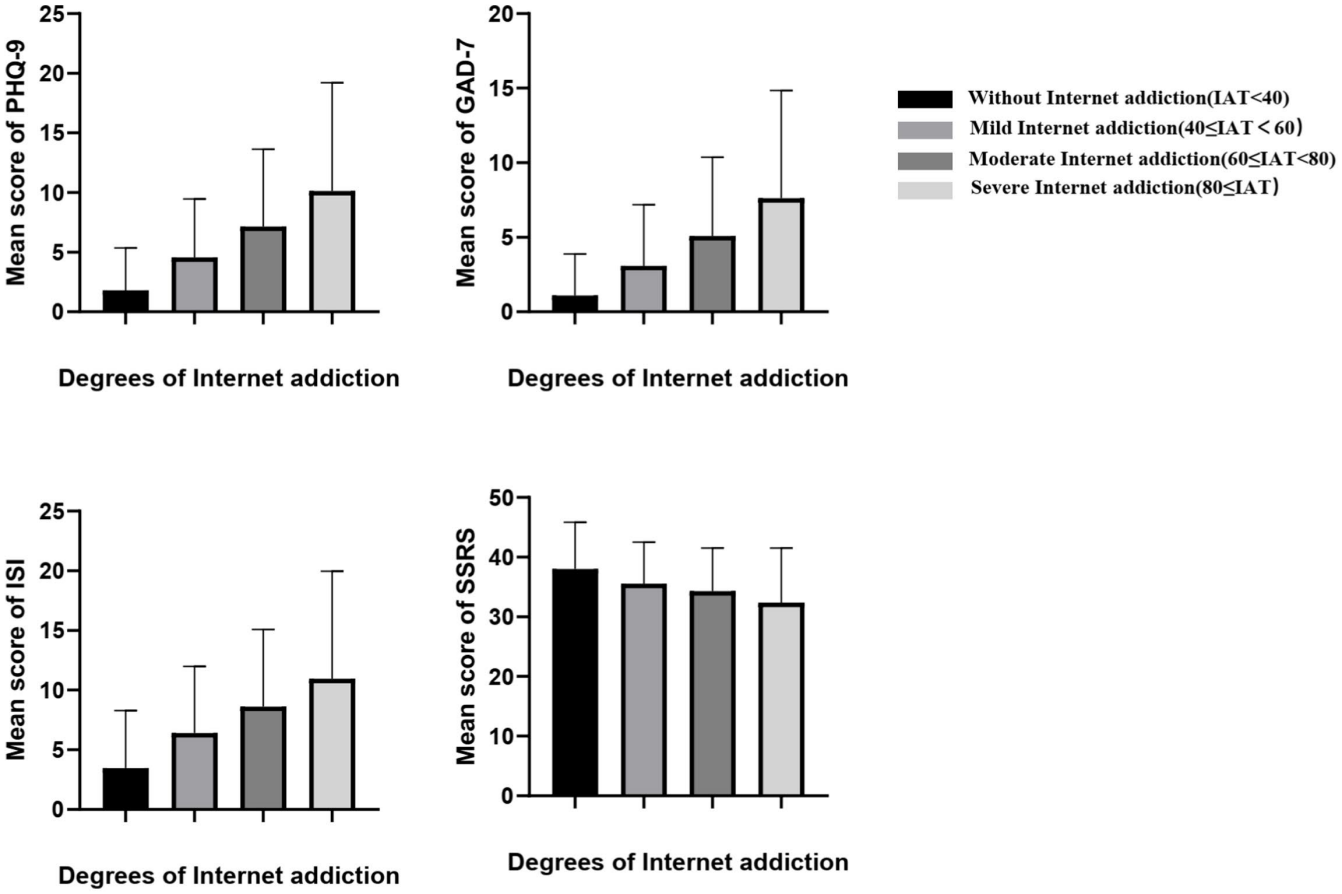
Comparison of mental health among different level of internet addiction. The figure showed that the higher the level of IA, the severe the depression symptom, anxiety symptom and sleep problem the participants reported, and the lower level of social support they received (*p* < 0.05).

As shown in Table 2, compared with participants with IA group, participants without IA group had significantly lower mean scores of PHQ-9 (*T* = −53.336, *p* < 0.001), GAD-7 (*T* = −48.479, *p* < 0.001), and ISI (*T* = −48.309, *p* < 0.001), higher mean scores of SSRS (*T* = 29.402, *p* < 0.001), and lower percentage of lifetime suicidal ideation (*χ^2^* = 504.815, *p* < 0.001). As shown in Figure 1, the higher the level of IA, the severe the depression symptom, anxiety symptom and sleep problem the participants reported, and the lower level of social support they received (*p* < 0.05).

**Table 2:** Comparison of mental health problems between the two groups.

	Total (*N* = 22,605)	Without IA (*N* = 7,684)	With IA (*N* = 14,921)	*T/χ^2^*
PHQ-9	4.45 ± 5.7	1.79 ± 3.569	5.82 ± 6.095	−53.336^a^***
GAD-7	3.06 ± 4.591	1.1 ± 4.07	4.07 ± 4.99	−48.479^a^***
ISI	6.11 ± 6.169	3.48 ± 4.795	7.46 ± 6.358	−48.309^a^***
SSRS	35.98 ± 7.624	38.02 ± 7.845	34.93 ± 7.288	29.402^a^***
Suicidal ideation	5,741 (25.4)	1,255 (16.3)	4,486 (30.1)	504.815^b^***

IA, internet addiction; ***, *p* < 0.001.

a Two-sample *t* test.

b Chi-square test.

### 3.3. Interacting effects of IA and academic satisfaction on mental health

**Figure 2:**
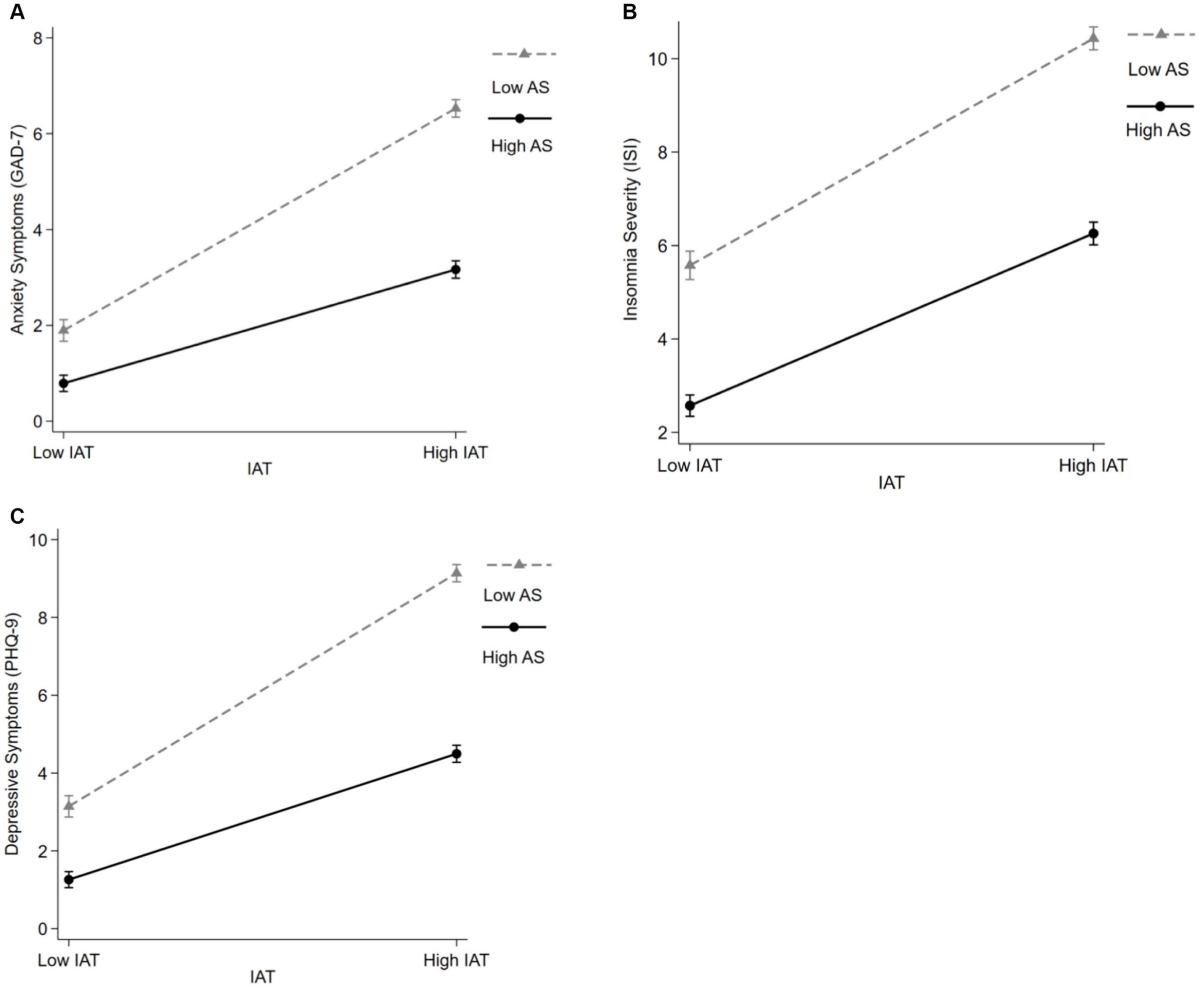
The interact impact of internet addiction and academic satisfactory on mental health problems. **(A)** An effect of IA on depression (*t* = 12.85, *p* < 0.001, *B* = 0.077, 95% *CI*: 0.065; 0.089), and a significant interaction between IA and academic satisfactory on depression (*t* = 10.27, *p* < 0.001, *B* = 0.021, 95% *CI*: 0.017; 0.025). **(B)** An effect of IA on anxiety (*t* = 11.1, *p* < 0.001, *B* = 0.05, 95% *CI*: 0.045; 0.064) and an effect of academic satisfactory on anxiety (*t* = −2.81, *p* = 0.005, *B* = −0.25, 95% *CI*: −0.45; −0.76). Significant interaction between IA and academic satisfactory on anxiety (*t* = 10.22, *p* < 0.001, *B* = 0.017, 95% *CI*: 0.014; 0.02). **(C)** An effect of IA on insomnia (*t* = 15.46, *p* < 0.001, *B* = 0.1, 95% *CI*: 0.09; 0.12) and an effect of academic satisfactory on insomnia (*t* = 3.96, *p* < 0.001, *B* = 0.48, 95% *CI*: 0.24; 0.71). Significant interaction between IA and academic satisfactory on insomnia (*t* = 3.93, *p* < 0.001, *B* = 0.008, 95% *CI*: 0.004; 0.013). AS, academic satisfactory.

As shown in Figure 2, an effect of IA was found on depression (*t* = 12.85, *p* < 0.001, *B* = 0.077, *95% CI:* 0.065–0.089), anxiety (*t* = 11.1, *p* < 0.001, *B* = 0.05, *95% CI*: 0.045–0.064) and insomnia (*t* = 15.46, *p* < 0.001, *B* = 0.1, *95% CI*: 0.09–0.12). Participants with higher score of IA had more serious mental health problem. Furthermore, an effect of academic satisfactory was found on anxiety (*t* = −2.81, *p* = 0.005, *B* = -0.25, *95% CI*: −0.450–0.760) and insomnia (*t* = 3.96, *p* < 0.001, *B* = 0.48, *95% CI*: 0.240–0.710). Participants with lower level of academic satisfactory had more serious symptom of anxiety and insomnia. However, there was no effect of academic satisfactory on symptoms of depression. There was significant interaction between IA and academic satisfactory on symptoms of depression (*t* = 10.27, *p* < 0.001, *B* = 0.021, *95% CI*: 0.017–0.025), anxiety (*t* = 10.22, *p* < 0.001, *B* = 0.017, *95% CI*: 0.014–0.02) and insomnia (*t* = 3.93, *p* < 0.001, *B* = 0.008, *95% CI*: 0.004–0.013).

## 4. Discussion

This study investigated the relationship between IA and mental health problems in college students after the lifting of COVID-19 restrictions in Sichuan, China. The study showed that college students with IA were more likely to have depression symptom, anxiety symptom, insomnia, and lifetime suicidal ideation than those without IA. Importantly, participants with higher level of IA would experience more severe mental health problems (e.g., depression, anxiety, insomnia, and suicidal ideation) and receive fewer social support. Further, the study revealed that participants with higher level of IA, combining with lower level of academic satisfaction were more likely to have depression symptom, anxiety symptom and insomnia.

Compared with those without IA, participants with IA were more likely to have depression symptom, anxiety symptom, insomnia, and lifetime suicidal ideation, which is consistent with previous studies (23, 29, 34). Evidence shows that IA is often comorbid with various mental health problems such as suicidal ideation (34), sleep problems (35, 36), depression, and anxiety (23, 37). Previous studies also reveal that IA, specifically addictive gaming, may be linked to diminished sleep quality and reduced sleep duration, subsequently leading to the sleep-related issues (38). Cognitive-behavioral model of IA (34) and the theory for problematic internet use (38) illustrate that individuals suffering from psychological distress (such as depression) may be more likely to be frustrated in real life, and they would choose turn to the internet to make them feel safe and comfortable. Thus, it is important to prevent IA and reduce psychological distress after the COVID-19 pandemic for maintaining normal social activities and functioning among college students.

The results of this study showed that college students with IA had significantly higher rate of family history of psychosis than those without IA, which is consistent with previous study (34). Evidence suggests that there is a significant association between parental depression and IA in young people (39). Compared to young people with mild IA or without IA, those with moderate to severe IA have three times higher risks of parents with moderate to severe depression (40). Parental depression may contribute to adolescent stress, which in turn can lead to adolescent internet addiction (40). Moreover, parent with IA may have a significant negative effect on their offspring (41, 42). Therefore, characteristics and mental health problems of parents should also be considered and addressed for education and psychosocial interventions for reducing IA and improving mental health of college students.

This study revealed that participants with higher level of IA would experience more severe mental health problems, which might be explained by the mechanism of IA. IA, a behavioral addiction, shares several similar traits with gambling disorder (33), and more importantly, it also shares the similar addictive patterns in neurobiology (43–45). Evidence has shown that IA would contribute to a release of dopamine at a rapid rate, which may lead to immediate gratification and the potential for a repetitive response, including compulsive behaviors and increased tolerance (46). In fMRI studies, dysregulated reward processing and diminished impulse control were found in people with IA (47). Thus, more serious IA might imply the development of organic pathologies, so youth with IA needs to early intervention and prevention in advance.

This is the first study to reveal the interaction between IA and academic satisfaction on mental health problems. The study showed that the higher degree of IA, combining with the lower academic satisfaction would contribute to mental health problems such as depression, anxiety, and insomnia. Previous studies indicated that IA could be seen as a maladaptive coping strategy to deal with problematic conditions of students (48). Those problematic conditions could include challenges in interpersonal interactions, such as difficulties in establishing and maintaining friendships (48) and dysfunctional familial relationships (18). And in this study, students with IA had higher level of academic unsatisfaction. Evidence shows that escapism by means of immersion has the strongest association with addictive behaviors (49). Therefore, this study suggests that students with IA may not only escape from the problematic relationships, but also from the failure of academic performance. On the other hand, IA has also impaired students’ academic satisfaction (18). IA and academic satisfaction might create a vicious cycle: IA might lead to the decrease of cognitive function (22) and academic procrastination (50, 51), and it would further lead to poor academic performance, which in turn lead to the escapism. Thus, to break this vicious cycle, it is necessary to prevent IA and improve students’ academic studies. More importantly, it is important to address the problems that cause the IA to escape into internet world, which means that the educational institutes, schools and parents need to help students with their academic performance.

### 4.1. Strengths and limitations

To our knowledge, this is the first study to explore the college students’ IA and the mental health problems after the lifting of COVID-19 restrictions in China. Also, this study has a large sample size of students in different colleges and universities from freshmen to senior students in Sichuan, China. The response rate of the survey was very high (92.0%). Moreover, the questionnaire of the survey was released to students directly through their teachers and professors, which may improve the quality of data collection.

This study also has a few limitations. This study was conducted only in Sichuan province, China, which may not generalize the results of college students to other areas of China. This study is a cross-sectional design, and no causal associations should be inferred. Further long-term follow-up studies on IA and mental health among college students should be conducted.

## 5. Conclusion

This initial study aimed to investigate the college students’ IA and the mental health problems during and after the COVID-19 pandemic. The results of this study showed that IA had a significant negative impact on the mental health problems among Chinese college students. The college students with IA were more likely to have depression, anxiety, insomnia, and suicidal ideation. And college students with serious IA would have more severe mental health problems (e.g., depression, anxiety, insomnia, and suicidal ideation) and lower level of social support. The study also firstly shows that IA and academic satisfactory have interact impacts on mental health problems among college students. Further educational and health policies and psychosocial interventions should be developed to reduce IA and enhance academic satisfaction for improving students’ mental health. Longitudinal studies should be conducted in the future to further clarify the causal relationship among IA, academic satisfaction and mental health problems.

## Data availability statement

The original contributions presented in the study are included in the article/supplementary materials, further inquiries can be directed to the corresponding author.

## Ethics statement

The studies involving humans were approved by Biomedical Research Ethics Committee of West China Hospital, Sichuan University (No: 2022-1790). The studies were conducted in accordance with the local legislation and institutional requirements. Written informed consent for participation in this study was provided by the participants’ legal guardians/next of kin.

## Author contributions

M-SR designed this study. M-SR, A-PD, JaC, Z-YD, CW, Y-FM, H-JS, YH, and WZ conducted this study. A-PD, CW, JaC, M-SR, and Z-YD conducted data analysis. A-PD, CW, JaC, and M-SR wrote the first draft of the paper. A-PD, CW, JaC, Z-YD, Y-FM, H-JS, Y-JM, X-DM, X-HH, LZ, YH, WZ, JnC, and M-SR participated in the data collection and made contributions to critical revision of the manuscript. All authors contributed to the article and approved the submitted version.
